# Heterogeneous leaves of predominant trees species enhance decomposition and nutrient release in the riparian zone of the Three Gorges Reservoir

**DOI:** 10.1038/s41598-020-74062-4

**Published:** 2020-10-15

**Authors:** Zhangting Chen, Chaoying Wang, Xuemei Chen, Zhongxun Yuan, Hong Song, Changxiao Li

**Affiliations:** 1 Key Laboratory of Eco-Environments in the Three Gorges Reservoir Region (Ministry of Education), State Cultivation Base of Eco-Agriculture for Southwest Mountainous Land, College of Life Sciences, Southwest University, Chongqing, 400715 China; 2 Guilin Tourism University, Guilin, 541006 China; 3 Chongqing City Management College, Chongqing, 401331 China

**Keywords:** Restoration ecology, Riparian ecology

## Abstract

The leaves of riparian plants are the main source of energy and nutrients in riparian ecosystems. In order to evaluate the nutrient release of reforested trees in a riparian zone, a field litterbag experiment involving three foliar types (the leaves of either coniferous and broadleaf trees as single-leaf treatment, or a mixture of coniferous and broadleaf leaves as a heterogenous-leaf treatment) and different submergence depths [no submergence (CK), shallow submergence (SS), and deep submergence (DS)] was conducted in situ in the Three Gorges Reservoir (TGR) for one year. The results showed that, when compared to the single-leaf treatment, the heterogenous-leaf treatment exhibited greater mass loss at both SS and DS, in contrast to a greater nitrogen release rate only at DS and a greater phosphorous release rate only at SS. Overall, submergence facilitated decomposition and nutrient release, although the decomposition rate was higher in SS than in DS. The results suggested that the decomposition and nutrient release of the three foliar types may increase the potential pollution risk to the TGR water environment. Thus, we propose that the leaves of the reforested riparian stands be harvested prior to submergence to preserve the water quality of the TGR.

## Introduction

The Three Gorges Dam (TGD) in the upper reaches of the Yangtze River is the largest dam in the world^1^. After the establishment of the TGD in 2003, a riparian zone covering an area of 349 km^2^ was created due to an annual periodic water level change from 145 to 175 m above sea level (a.s.l.) in the Three Gorges Reservoir (TGR)^2^. This artificial water regime in the TGR has led to long-term periodic deep submergence in the riparian zone, with the flooding time and duration being opposite to the natural hydrological regime of the Yangtze River. Since most riparian plants cannot tolerate these great water level changes, losses of many intolerant riparian plants in the TGR have caused severe environmental degradation of the riparian zone^1,3,4^. Moreover, the continuous degradation of the riparian zone environment has caused a functional decline in the riparian ecosystem, thus further increasing the potential pollution risk to the TGR water environment. Currently, the decreasing water quality has become a major environmental concern in the TGR^5,6^. Studies have shown that algal blooms frequently develop in the tributary backwaters of the TGR due to the accumulation of nutrients [e.g., nitrogen (N) and phosphorus (P)] triggered by these water level changes in the riparian zone^7,8^. Riparian vegetation restoration is a crucial step for addressing the abovementioned environmental issues of the TGR^9,10^. At present, artificial vegetation restoration is being carried out, and many monocultures and mixed coniferous–broadleaf restoration plantations are still to be reforested in the riparian zone of the TGR^11–13^. These reforested plantations not only affect the water quality but also influence nutrient cycling within the TGR^14,15^. Of the riparian species used in the restoration plantations, bald cypress (*Taxodium distichum*), pond cypress (*Taxodium ascendens*), and Chinese willow (*Salix matsudana*) are predominantly planted in the riparian zone because of their strong flooding tolerance abilities. However, little is known about the differences in the foliar decomposition and nutrient release of monocultures versus mixed plantations during submergence under the planting of coniferous and broad–leaved tree species. This may be closely linked to the water quality of the TGR, thus having implications for the riparian forest management of the riparian zone.

Foliar decomposition and nutrient dynamics are fundamental ecological processes of the riparian ecosystem that are regulated by environmental conditions, leaf quality, and decomposers^16–18^. When the water level rises to 175 m.a.s.l. in the riparian zone, the submerged perennial trees, such as the aforementioned deciduous species, will decompose and release large amounts of nutrients, e.g., N and P, which may both directly and indirectly deteriorate the water quality in the TGR^2,4,19^. However, as the effects of mixed decomposition and nutrient cycling of the mixed plantations are poorly understood, this hinders the scientific management of reforested riparian plantations and precludes an accurate prediction of the impact of mixed riparian vegetation in the riparian zone on the water quality of the TGR.

Prior studies have shown that, due to the interactions between different species in the natural environment, mixed decomposition cannot always be predicted from single species dynamics^20–22^. Theoretically, because more than one resource type is present, nutrients (e.g., N, P) can be transferred from leaves with high nutrient concentrations to adjacent with lower nutrient concentrations^23,24^. The decomposers of nutrient-poor leaves could thus be enhanced and stimulated, or conversely could be suppressed by the release of potential inhibitory compounds, such as phenolics and tannins. Richer decomposers per unit of mixed species mass can typically be supported^23^. In addition, the mixtures of different species with varied chemical and physical characteristics may not only provide a heterogeneous microenvironment, but may also supply food resources and alter the physical breakdown or abrasion processes of low-quality species, which ultimately affects the decomposition of all samples^25–28^. All the above-mentioned mechanisms lead to differences in decomposition between single- and mixed-leaf compositions. Moreover, differences in the aquatic and terrestrial decomposition environments, as well as differences between water temperature, water depth, and dissolved oxygen in the aquatic environment, will affect the decomposition rate^29^. However, the process of decomposition and nutrient release of different leaf mixtures under different water submergence scenarios is unclear. Hence, it is necessary to study the decomposition and nutrient release of mixed plantations in the context of an artificial hydrological regime, which can enhance our understanding of the riparian ecological environment of the TGR.

Studies have revealed that submergence can promote the decomposition and increase the nutrient release of a single leaf type in the riparian zone of the TGR^30–32^. Thus, we hypothesized that: (1) a mixture of leaves from both coniferous and broadleaf species might have different decomposition and nutrient release properties to leaves from single leaf types due to differences in composition and concentration; and (2) prolonged deep-water submergence may reduce foliar decomposition and nutrient release due to the lack of dissolved oxygen and other nutrients conducive to decomposition. To test these assumptions, a field decomposition experiment including three foliar types (leaves of either coniferous or broadleaf species as single-leaf treatment and a mixture of coniferous and broadleaf species leaves as a heterogenous-leaf treatment, in equal mass proportions) and three water submergence depths [no submergence as a control check (CK), 0.5 m shallow submergence (SS), and 5.0 m deep submergence (DS)] was conducted in situ in the riparian zone of the TGR throughout an entire year (i.e., a hydrological cycle). We measured the mass loss and nutrient release of C, N, and P across a period of one year. The objectives of this study were to determine: (1) the effects of heterogenous-leaf treatment on the decomposition rate of the reforested trees in the riparian zone of the TGR, and (2) the effects of water depth on the decomposition and nutrient release rate of three foliar types (single coniferous, single broadleaf, and mixed coniferous and broadleaf) in the TGR. The findings of this study can provide a theoretical foundation for the best management practices for reforested tree species in the artificial hydrological regime of the TGR in China.

## Results

### Foliar mass loss

Foliar mass loss generally increased over time but varied between treatments and incubation times. Overall, there were significant effects in terms of the remaining mass by foliar type, water treatment, decomposition time, and their interactions (Table 1). Over the 360 days of decomposition, the single- and heterogenous-leaf treatments lost 83–90% of their initial dry mass in SS and 79–85% in DS, compared to 54–67% in CK (Table 2). Obviously, foliar decomposition was significantly promoted in both SS and DS throughout the experimental period, with the mean mass loss being 31.22% and 28.09% higher than that in CK, respectively (Table 2 and Fig. S1). Furthermore, the foliar decomposition rate in SS was significantly higher by 3.13% than that in DS, regardless of foliar type (F = 0.164, *p* < 0.01). Throughout the experiment, greater rates of foliar decay occurred during the first 30 days, followed by a decline as decomposition proceeded (Table 2 and Fig. S1).

**Table 1:** Repeated-measures ANOVA results for the remaining foliar mass and contents of C, N, and P.

Source of variation	F-value			
	MR	C	N	P
Foliar type	281.22***	63.69***	89.16***	124.76***
Submergence depth	3398.32***	1465.28***	450.05***	545.32***
Decomposition time	2713.44***	1288.96***	457.16***	572.17***
Foliar type × submergence depth	16.69***	14.12***	0.81	6.387**
Foliar type × decomposition time	8.39***	6.70***	5.75***	6.22***
Decomposition time × submergence depth	66.93***	32.54***	24.25***	28.50***
Foliar type × decomposition time × submergence depth	4.44***	4.03***	1.68*	1.32

MR, Mass remaining.

*p < 0.05, **p < 0.01, ***p < 0.001.

**Table 2:** Mass loss and nutrient release of C, N, and P after 30 days and 360 days decomposition of bald cypress (A), Chinese willow (B), and heterogeneous-leaf (C) treatments under different submergence depths.

Depth	Foliar type	Mass loss (%)		C release (%)		N release (%)		P release (%)	
		30 days	360 days	30 days	360 days	30 days	360 days	30 days	360 days
CK	A	18.95 ± 1.07Cb	53.71 ± 0.4Cc	16.48 ± 1.26Bb	71.44 ± 2.39Ab	− 11.62 ± 6.94Bb	53.92 ± 3.29Ab	22.06 ± 2.77Ab	85.2 ± 0.5Ac
	B	27.81 ± 0.76Ac	67.08 ± 1Ac	28.13 ± 1.94Ab	73.05 ± 0.79Ac	− 1.26 ± 2.84ABb	53.64 ± 0.91Ac	19.59 ± 6.23Ab	77.33 ± 0.47Bb
	C	23.55 ± 1.16Bb	61.43 ± 0.74Bc	25.63 ± 1.59Ab	64.87 ± 0.27Bc	10.17 ± 2.97Ab	60.22 ± 1.98Ab	24.68 ± 4.96Ab	70.4 ± 1.97Cb
SS	A	50.68 ± 0.61Ba	82.89 ± 0.48Ba	42.01 ± 1.06Ba	86.5 ± 0.55Ba	29.1 ± 3.19Ba	76.58 ± 0.59Ba	68.59 ± 0.57Ca	90.82 ± 0.25Bb
	B	63.83 ± 0.35Aa	89.05 ± 1.77Aa	55.36 ± 0.54Aa	91.87 ± 1.33Aa	29.84 ± 2.5Ba	87.98 ± 1.45Aa	86.45 ± 0.5Aa	92.99 ± 1.04Ba
	C	62.05 ± 1.82Aa	90.09 ± 0.32Aa	55.69 ± 2.13Aa	90.15 ± 0.35Aa	40.06 ± 2.32Aa	86.72 ± 1.02Aa	77.8 ± 0.54Ba	96.23 ± 0.5Aa
DS	A	48.23 ± 1.39Ca	79.32 ± 1.37Bb	43.3 ± 3.33Ba	82.69 ± 1.74Aa	28.47 ± 1.04Ba	77.73 ± 2.96ABa	63.76 ± 1.61Ba	92.77 ± 0.55Aa
	B	59.29 ± 0.65Bb	78.84 ± 0.59Bb	53.39 ± 1.28Aa	79.58 ± 1.55Ab	26.97 ± 2.87Ba	74.09 ± 2.14Bb	81.98 ± 0.98Aa	93.65 ± 0.29Aa
	C	64.42 ± 0.76Aa	84.96 ± 1.76Ab	58.68 ± 0.93Aa	85.69 ± 2.19Ab	46.78 ± 2.3Aa	83.99 ± 2.15Aa	78.92 ± 1.12Aa	94.63 ± 0.9Aa

Different capital letters indicate significant differences in mass loss and nutrient release from different foliar types under the same treatment; different lowercase letters indicate significant differences in mass loss and nutrient release of the same foliar type under different treatments.

CK, control check (no submergence); SS, shallow submergence, DS deep submergence.

**Figure 1:**
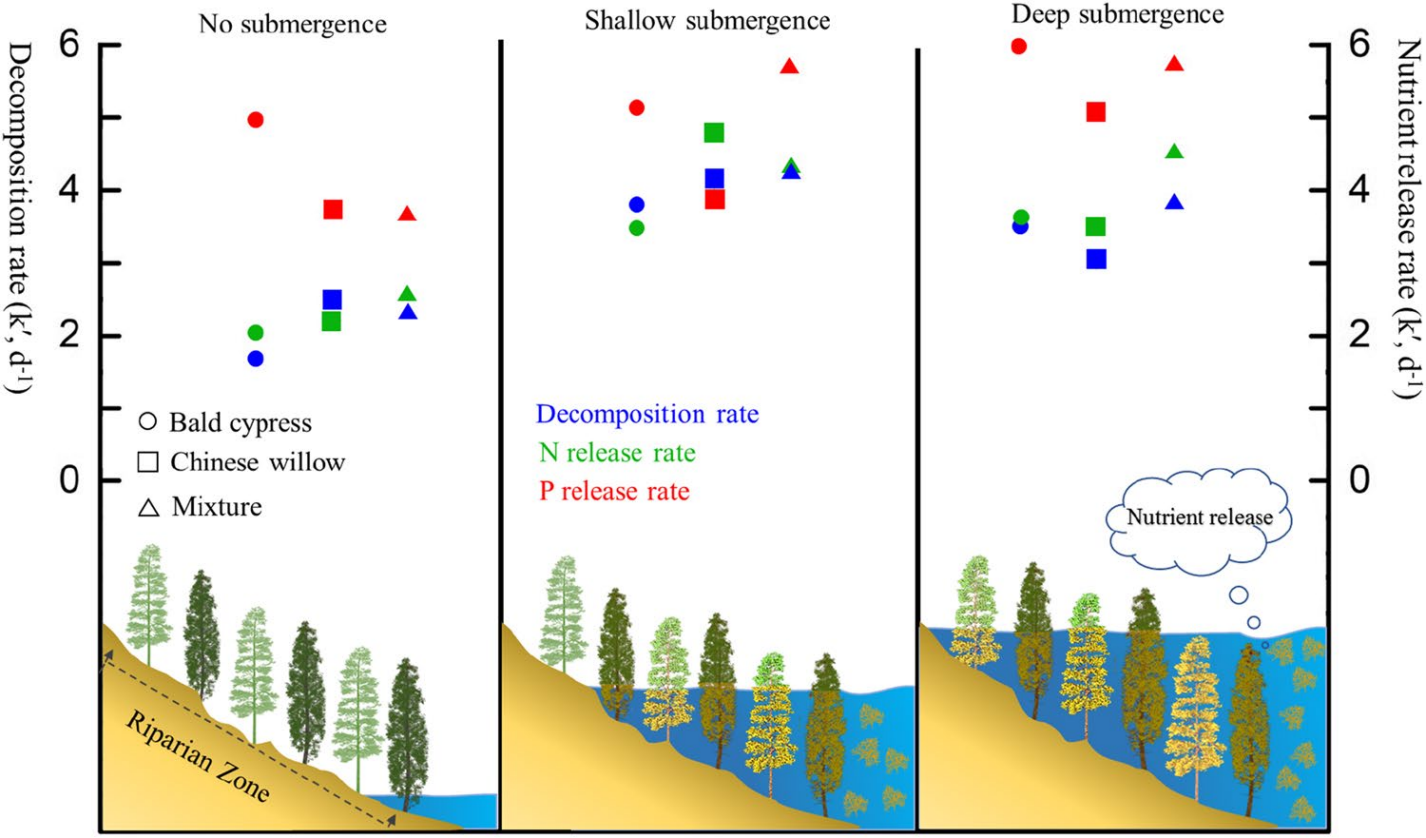
Changes in the decomposition and nutrient release rate (*k*, g g^−1 ^days^−1^) of the leaves of bald cypress, Chinese willow, and the mixture under different submergence depths.

The decomposition rates were not consistent across all foliar types under different water gradients (F = 299.938, *p* < 0.001), ranging from 0.00380 to 0.00425 days^−1^ in SS and 0.00304 to 0.00383 days^−1^ in DS. In contrast, a significantly lower level of 0.00170 to 0.00247 days^−1^ was recorded in CK (Table 3). The *k*-value of the leaves of bald cypress, the leaves of Chinese willow, and a mixture thereof under SS was 2.24, 1.68, and 1.85 times that under CK, respectively. Likewise, the *k*-value of the leaf of bald cypress, leaf of Chinese willow, and the mixture under DS was 2.07, 1.23, and 1.67 times that under CK, respectively. However, each foliar type in SS had significantly higher *k*-values when compared to the corresponding values in DS (F = 568.157, *p* < 0.001). Furthermore, the *k*-value of the heterogenous-leaf treatment was significantly higher than that of the single-leaf treatment under SS and DS submergence treatments (both *p* < 0.001), with the *k*-value being 0.0011–0.0045 days^−1^ and 0.0031–0.0079 days^−1^ higher than that in SS and DS, respectively (Fig. 1 and Table 3). According to the Olson exponential model, the time required to decompose 95% of the samples in SS was the shortest (approximately 704–788 days), in contrast to a much longer time period in DS (approximately 782–985 days) and in CK (approximately 1212–1762 days) (Table 3).

**Table 3:** Equations and parameters of the natural logarithm (y) of the remaining leaf mass regressed against the decomposition days (*t*) of bald cypress (A), Chinese willow (B), and heterogeneous-leaf (C) treatments under different submergence depths (n = 40).

Depth (m)	Foliar type	Regression models	k′	R^2^	*p *value	t_0.95_ (days)
CK	A	y = 0.889e^−1.70t^	1.70	0.88	< 0.001	1762.20
	B	y = 0.837e^−2.47t^	2.47	0.91	< 0.001	1212.85
	C	y = 0.831e^−2.30t^	2.30	0.92	< 0.001	1302.49
SS	A	y = 0.640e^−3.80t^	3.80	0.80	< 0.001	788.35
	B	y = 0.523e^−4.14t^	4.14	0.74	< 0.001	723.61
	C	y = 0.509e^−4.25t^	4.25	0.74	< 0.001	704.88
DS	A	y = 0.640e^−3.52t^	3.52	0.81	< 0.001	851.06
	B	y = 0.545e^−3.04t^	3.04	0.69	< 0.001	985.44
	C	y = 0.520e^−3.83t^	3.83	0.70	< 0.001	782.18

t_0.95_, time of 95% decomposition; k′, k × 10^3^; CK, control check (no submergence); SS, shallow submergence; DS, deep submergence.

### Foliar nutrient release

The initial C and P contents of the bald cypress leaves were significantly higher than that of Chinese willow. On the contrary, the initial N content of the bald cypress leaves were significantly lower than that of Chinese willow. The initial content of C, N, and P and the ratios of C/N and N/P of the heterogenous-leaf treatment was not higher than the highest values or lower than the lowest values of the single-leaf treatment (Table S1).

The repeated-measures ANOVA revealed that the contents of C, N, and P were significantly affected by foliar type, water treatment, decomposition time (all *p* < 0.001 for C, N, and P), and their interactions (all *p* < 0.05, except for the interaction of foliar type × water treatment for N and interaction of foliar type × water treatment × decomposition time for P) (Table 1). The nutrient release of all foliar types exhibited an increasing trend over time but differed among water treatments and incubation times (Fig. S2). During the entire period of experimentation, the foliar nutrient release rates of C, N, and P were significantly higher in both SS and DS as compared to that in CK, regardless of foliar type (with the exception of C of Chinese willow in DS), although the effects of different submergence depths differed between foliar types (Table 4 and Fig. S2). Furthermore, across the three foliar types, the *k*-value of C release rate was always highest in SS, whereas the P release rate was greatest in DS. Interestingly, the *k*-value of the N release rate was highest in SS in Chinese willow, but was highest in DS in both the bald cypress and the heterogenous-leaf treatment. In comparison to SS, DS significantly accelerated P and N release rate (except for the N release of Chinese willow) while inhibiting the C release rate of all three foliar types (Table 4).

**Table 4:** Changes in the nutrient release rate (*k*, g g^−1^ days^−1^) of bald cypress (A), Chinese willow (B), and heterogeneous-leaf (C) treatments under different submergence depths.

Foliar type	Depth (m)	C		N		P	
		*k*′	R^2^	*k*′	R^2^	*k*′	R^2^
A	CK	2.97	0.87	2.03	0.73	4.69	0.87
	SS	4.84	0.87	3.48	0.80	5.13	0.79
	DS	3.75	0.73	3.61	0.77	5.96	0.84
B	CK	3.21	0.87	2.19	0.86	3.73	0.87
	SS	5.07	0.82	4.77	0.87	3.92	0.44
	DS	3.21	0.64	3.49	0.73	5.08	0.66
C	CK	2.79	0.87	2.53	0.86	3.65	0.75
	SS	4.66	0.83	4.30	0.87	5.67	0.70
	DS	4.31	0.75	4.50	0.80	5.72	0.74

k′, k × 10^3^; CK, control check (no submergence); SS, shallow submergence; DS, deep submergence.

Throughout the experimental period, most of the nutrient release of the heterogenous-leaf treatment was greater than that of the single-leaf treatment, despite a few differences on certain sampling dates (*p* > 0.05) (Fig. S2). Compared with the overall mean of these nutrients released from the single-leaf treatment, the effect of heterogenous-leaf treatment increased the foliar C, N, and P release by 4.42%, 10.8%, and − 0.23% for CK, 4.66%, 7.19%, and 2.14% for SS, and 4.98%, 6.67%, and 0.75% for DS, respectively (with the exception of P in CK). Furthermore, the nutrient release rates (C, N, P) of all the three foliar types were significantly correlated with their mass loss rates (*p* < 0.01) (Table S2).

## Discussion

Foliar decomposition is a fundamental biogeochemical process that plays a critical role in regulating nutrient turnover in terrestrial ecosystems^23^. In general, the process of decomposition in terrestrial environments has two phases, namely the early phase and the late phase. In the early phase, up to 20–40% of the total mass of the leaf samples can be lost, which is largely modulated by leaf chemistry^33^. In the late phase, the remaining mass of the leaf samples is decomposed, which is largely influenced by microbial community^34^. Unlike terrestrial conditions, there are many different factors under aquatic conditions, such as dominant detritivores, physical abrasion, water availability, and temperature range, that are known to directly and indirectly affect foliar decomposition^35–37^. In our study, the mass loss of all foliar types was greatest during the first 30 days of decomposition, regardless of foliar type (Fig. S1), and the rate of decomposition under submergence in both SS and DS was much faster than that under the terrestrial environment in CK (Table 3). This was mainly attributed to the stronger flushing and leaching effects of the lotic environment, which increased the physical fragmentation of the leaves relative to that of the terrestrial environment^37^. In general, the decomposition rate in terrestrial environments is lower than that in aquatic conditions due to the lower water availability in terrestrial conditions^38^. More importantly, low water levels in terrestrial environments limit the development and growth of decomposers as well as the leaching of soluble chemical compounds during leaf decomposition phase expriments^39,40^.

Throughout the entire period of experimentation, there was a significant difference in foliar decomposition rate between SS and DS (Table 3), indicating that different submergence depths could provide different decomposition conditions and thus result in a varied mass loss of different foliar types^29,41^. Compared to DS, there were better decomposition conditions under SS, such as higher dissolved oxygen and water temperature (Fig. S3), and the increase in water temperature is likely to have stimulated decomposer communities and their activities, all of which directly promoted the mass loss of each foliar type^38,42^. Furthermore, the relatively higher water temperature itself is also a vital factor facilitating the mass loss^43^.

In the current study, we found that the heterogenous-leaf treatment decomposed faster than the single-leaf treatment in both SS and DS (Table 3), indicating that mixing of the leaves of both bald cypress and Chinese willow accelerated the decay. Some studies have reported that mixtures of coniferous-broadleaf species typically speed up decomposition rates in forests^44,45^. In general, leaf mixtures often have different physical and chemical features from single leaf types alone, thus creating a heterogenous and complex environment with diverse physical habitats and nutritional resources, leading to an overall improvement in decomposer abundance and activity. In return, such improved decomposer abundance and activity can further stimulate decomposition rates on leaf mixtures^46–48^. However, our experiments only found that under the submerged environments of SS and DS, the decomposition rate of the heterogenous-leaf treatment was faster than that of the single-leaf treatment (Table 3). This was mainly related to leaf quality, habitat characteristics, and incubation time^22,25,35,37,41^. Therefore, our results support our first hypothesis that heterogenous-leaf treatment would have a different decomposition rate than a single leaf type. In the TGR, the higher leaf mass loss rates of the heterogenous-leaf treatment under SS and DS were associated with the combined effects of such factors as mentioned above.

Environmental factors that affect the decomposition of leaves may also indirectly influence the release of nutrients^49^. For example, oxygen levels have been found to differ between aquatic and terrestrial environments, resulting in different levels of decomposition by decomposers in these two systems^35^, which was also observed in our experiment (Fig. S3). Under SS and DS submergence conditions, the concentrations of foliar nutrients and the microbial biomass were found to decrease compared to that under terrestrial conditions, which implies that flow transport and cycling may lead to rapid reductions in nutrient concentrations in submerged rather than non-submerged conditions^37,46^. Thus, the nutrients in single- and heterogenous-leaf environments have been found to be somewhat more stable in terrestrial ecosystems than in aquatic environments. Moreover, stream fungi were found to be relatively more efficient in decomposing leaves compared to terrestrial fungi^50^, despite the fact that there is a higher microbial biomass in terrestrial leaves in general. However, terrestrial microbes have a higher capacity to immobilize and retain nutrients in the leaves, as they are less likely to have hydrologic flow limitations. This was supported by our experimental results, as after a year of decomposition, the nutrient residue of each foliar type that decomposed on land was significantly greater than that in the water (Fig. S2). Overall, leaf quality, environmental situations, and selective aquatic decomposers are primarily responsible for the differences in foliar nutrient release under submergence^18,29,35,43^.

In this study, we found that the nutrient release rate in the heterogenous-leaf treatment varied greatly with time (Table 1), which is in accordance with previous studies^26,47,51^. Such a variation can be mainly attributed to alterations in decomposer community, leaf chemical components, and microclimate. As decomposition progressed, the effects of leaf mixtures heterogeneity were intensified, possibly due to the successional changes in the dominant foliar consumers (e.g., fungi, detritivores, or bacteria) that synergistically participate in the decomposition of leaf mixtures^18,35^. However, while fungal biomass and breakdown rates are positively correlated for single-leaf environments in streams,^52^ the mechanisms linking the diversity, bacterial and fungal biomasses, and nutrient release of heterogenous-leaf treatment remain unclear and should be investigated further.

The rate at which the three foliar types released their nutrients differed significantly with submergence depth (Fig. 1 and Table 4). The rates of N release from the three foliar types improved with submergence depth (except N release in submerged Chinese willow) in our study, which provides another indication that deeper and prolonged submergence stimulates the release of foliar N nutrient. This partly coincides with the findings of Xie^29^. In particular, the N and C levels in heterogenous-leaf treatment was altered remarkably compared to the single, and the nutrient release rate of N and C of the heterogenous-leaf treatment was greater than that of the single under DS (Fig. S2 and Table 4), indicating that the C and N cycles as well as the transfer of nutrients in mixtures are highly dynamic, and furthermore, that heterogenous-leaf treatment could promote the decomposition of matter and release of nutrients to the water body. The form of P in the leaves exists as a bioactive element, making it more readily available for release^51^. P can abruptly release into the water during the process of decomposition^53^. This is supported by our study, as P release was much faster than N and C release in all three foliar types (Table 4). Moreover, the P release also increased with submergence depth, and the nutrient release rate of P of the heterogenous-leaf treatment was greater than that of the single-leaf treatment under SS (Table 3). Fraser^54^ reported that P can be released via migration and leaching from plants, as it exists in the form of phosphate anions or compounds. Thus, our second hypothesis was not validated in terms of the N and P release dynamics of the leaf samples in the present study. In addition, the N and P concentrations of plants are key factors that influence the decomposition, and the changes in the concentrations of these elements alter the release of nutrients^32,55^.

The decomposition and nutrient release rates of single- and heterogenous-leaf environments are influenced by several factors, such as the type of plants, leaching methods, humidity, temperature, incubation time, and other physical conditions^23^. In our study, the nutrient release rate of each foliar type had a significant positive correlation with mass loss (Table S2), indicating that the greater the mass loss of tree leaves, the more nutrients they release. These results show that the release of nutrients (e.g., N and P) from the leaves may cause some level of nutrient loading in the water environments of the TGR. Therefore, the adequate management of coniferous–broadleaf dominant plantations, such as the sustainable harvesting of branches and leaves before they are submerged each year, should be considered in the TGR.

## Conclusions

The findings of this study show that coniferous-broadleaf leaf mixtures have a profound influence on the dynamics of decomposition. Specifically, a heterogenous-leaf environment strongly influences nutrient release and mass loss, which may further affect the primary productivity and nutrient cycling of riparian ecosystems. In the TGR, prolonged submergence promoted the decomposition of leaves and the release of nutrients from the three foliar types. The decomposition of the heterogenous coniferous–broadleaf treatment was faster than that of the single-leaf treatment, and the nutrient release rate of N and P was also faster at DS and SS, respectively. We found that the decomposition of the three foliar types was dependent on leaf quality and abiotic factors (e.g., duration and depth of submergence). Considering that most reservoirs will cause a certain degree of vegetation submergence in the riparian zone, the plant nutrients released into the water body will have a potential negative impact on the water quality. Thus, effective management approaches should be considered, such as harvesting the branches and leaves from the vegetation prior to submergence. These approaches will reduce the leaf biomass available for decomposition and therefore alleviate the deterioration of the water quality in reservoirs.

## Materials and methods

### Study site

The study was carried out in a riparian zone revegetation demonstration site, located in the Ruxi River Basin of the TGR in Zhong County, Chongqing Municipality, China (30° 24′ 16″ to 30° 24′ 56″ N; 108° 08′ 03″ to 108° 08′ 21″ E). This region has a subtropical southeast monsoonal climate, with an annual mean temperature of 18.2 °C and annual mean precipitation of 1200 mm. The soil in this region is classified as purple soil (Regosols in FAO Taxonomy or Entisols in USDA Taxonomy). The riparian forest is dominated by seven-year-old saplings of bald cypress, pond cypress, and Chinese willow, including both monocultures and mixed plantations.

### Experimental design

The foliar decomposition experiment was conducted in situ in the riparian zone. Given that the riparian water level across the riparian zone dynamically changes throughout the year, foliar submergence treatments were carried out in a small artificial reservoir (250 m long, 60 m wide, and 12 m deep) covering an area of over 700 m^2^ in order to effectively control the water level fluctuation during the entire period of experimentation. This artificial reservoir is closely connected to the riparian zone of the TGR, with its water being impounded directly from the TGR. The experiment was synchronized with the actual water level rise in the riparian zone of the TGR.

The decomposition experiment was conducted using the litterbag method. As bald cypress and pond cypress belong to the same genus and share many common traits, our experiment selected bald cypress as the coniferous representative and Chinese willow as the broad-leaved representative, as these two species have large differences in foliar size. In September 2017 when the TGR began to impound, fresh leaves from 12 trees of each species of bald cypress and Chinese willow exhibiting the same growth conditions in the riparian zone of the study site were collected and weighed. Each nylon litterbag (20 × 20 cm with a 0.25-mm mesh size) contained 15 g fresh leaves, with equal masses of each foliar type (15 g of bald cypress leaves, 15 g of Chinese willow leaves, and 7.5 g of bald cypress + 7.5 g of Chinese willow). The litterbags of each foliar type were then randomly divided into three groups for the different water submergence treatments of CK, SS, and DS. In order to monitor the dynamics of foliar decomposition and nutrient release, 10 sampling dates were designed, with four replicates of each foliar type under each water treatment per sampling date. Thus, there were a total of 360 litterbags (3 foliar types × 3 water treatments × 4 replications × 10 sampling dates). To determine the initial dry weight and initial leaf nutrient traits, we prepared an additional 12 litterbags for each of the three foliar types. The experiment lasted for a year from September 25, 2017 through to September 26, 2018. Litterbags were retrieved after 30, 60, 90, 120, 180, 210, 240, 270, 300, and 360 days of decomposition. Across the experimental period, the air and water temperature and dissolved oxygen content of the reservoir were concurrently recorded at each sampling date using a Hydrolab DS5 water quality multi-parameter monitor (Hydrolab, HACH, USA) (Fig. S3).

The collected litterbags were kept separate and placed into sealable plastic bags with ice and returned to the laboratory within 18 h. In the laboratory, the samples were gently rinsed in deionized water to remove any remaining sediment or invertebrates. The leaf samples were then transferred to pre-weighed paper bags, dried in an oven at 60 °C until a constant weight was achieved, and then reweighed to determine the mass. The dry leaf samples were ground into a powder, and the nutrient contents of C, N, and P were tested. The contents of C and N were determined using a CHNS-O elemental analyzer (CHNS-O-Vario EL cube, Heraeus Elementar, Hanau, Germany). The content of P was tested using inductively coupled plasma mass spectrometry (ICP-OSE) following heat digestion with a nitric acid and hydrogen peroxide mixture in a microwave digestion system (Speed Wave MWS-4). All the chemical analyses were performed in the laboratory at 20 °C.

### Statistical analysis

The mass loss and the nutrient release rate during each period of the experiment were calculated using the following equations^41^:

$${\text{D}}_{{\text{t}}} \left( \% \right) = ({\text{W}}_{0} - {\text{W}}_{{\text{t}}} )/{\text{W}}_{0} \times 100\%$$

$${\text{R}}_{{\text{t}}} \left( \% \right) = ({\text{W}}_{0} {\text{C}}_{0} - {\text{W}}_{{\text{t}}} {\text{C}}_{{\text{t}}} )/{\text{W}}_{0} {\text{C}}_{0} \times {1}00\%$$

The decomposition rate k was calculated using the exponential decay model^56^:

$${\text{W}}_{{\text{t}}} /{\text{W}}_{0} = {\text{ae}}^{{ - {\text{kt}}}} ,$$

where D_t_ and R_t_ are the mass loss rate (%) and the nutrient release rate (%); W_0_ and W_t_ are the initial foliar weight and the weight of the foliar remaining at time *t*; C_0_ and C_t_ are the initial nutrient concentration of the leaf samples (g kg^−1^) and the nutrient concentration of the leaf samples at time *t* (g kg^−1^). *k* (days^−1^) is the decomposition rate, *t* is the time of collection, and *a* is a constant. The time required for foliar decomposition at 95% is calculated by the equation t_0.95_ = ln0.05/(− k).

### Statistical analyses

Repeated-measures analysis of variance (ANOVA) was used to test the differences in foliar decomposition rates and changes in nutrients (C, N, and P) among the three foliar types (bald cypress, Chinese willow, and a mixture thereof) and three water submergences (CK, SS, and DS) over one year. A two-way ANOVA was conducted with decomposition time as the within-subject factor, and foliar type and submergence depth as between-subject factors. One-way ANOVA was used to analyze the influence of different water treatments at the same sampling time on mass loss and nutrient release, and Duncan's Multiple Range test was used to test for significance (α = 0.05). Pearson’s correlation analysis was used to analyze the relationship between the mass loss and nutrient release rate of each foliar type. In addition, exponential regression was used to fit the index of mass loss and nutrient release among treatments to decomposition time.

All analyses were performed in SPSS 22.0 (IBM Corp., Chicago, USA) and Excel 2007 for Windows. Figures were illustrated in Origin 8.5 (Origin Lab Corp., USA). Data in the text are shown as mean ± standard error (SE).

## Supplementary information

Supplementary Information.
