# Supplementary material for: Heterogeneous leaves of predominant trees species enhance decomposition and nutrient release in the riparian zone of the Three Gorges Reservoir
Source: Sci Rep. 2020 Oct 15;10:17382. doi: 10.1038/s41598-020-74062-4 (PMC7562734; doi:10.1038/s41598-020-74062-4)
Supplement: Supplementary file 1 — Supplementary Information. [file 41598_2020_74062_MOESM1_ESM.doc]

**Supplementary information**

**Heterogeneous leaves of predominant trees species enhance decomposition and nutrient release in the riparian zone of the Three Gorges Reservoir**

Zhangting Chen^1, 2^, Chaoying Wang^3^, Xuemei Chen^1^, Zhongxun Yuan^1^, Hong Song^1^, Changxiao Li^1^*

**Complete affiliations:**

^1^Key Laboratory of Eco-Environments in the Three Gorges Reservoir Region (Ministry of Education), State Cultivation Base of Eco-agriculture for Southwest Mountainous Land, College of Life Sciences, Southwest University, Chongqing 400715, China

^2^Guilin Tourism University, Guilin 541006, China

^3^Chongqing City Management College, Chongqing 401331, China

**Corresponding author:**

Changxiao Li

College of Life Sciences

Southwest University

Chongqing, 400715, China

Tel: 0086-23-68252365

E-mail: [lichangx@swu.edu.cn](mailto:lichangx@swu.edu.cn)

**Table S1. Initial foliar chemical characteristics of bald cypress (A), Chinese willow (B), and heterogeneous-leaf (C) treatments (mean ± SE, n = 4).**

| Foliar type | C (g·kg^−1^) | N (g·kg^−1^) | P (g·kg^−1^) | C/N | C/P | N/P |
| --- | --- | --- | --- | --- | --- | --- |
| A | 470.38 ± 2.82a | 16.05 ± 0.13c | 2.56 ± 0.03a | 28.86 ± 0.53a | 187.32 ± 4.39b | 6.26 ± 0.11c |
| B | 423.85 ± 0.34c | 20.90 ± 0.20a | 2.05 ± 0.03b | 20.09 ± 0.18c | 209.75 ± 2.07a | 10.23 ± 0.21a |
| C | 450.05 ± 0.84b | 19.78 ± 0.36b | 2.13 ± 0.03b | 22.92 ± 0.58b | 213.97 ± 3.57a | 9.31 ± 0.16b |

C: total organic carbon; N: total nitrogen; P: total phosphorus. Different lowercase letters indicate a significant difference between species for the same variable at *P* < 0.05.

**Table S2.** Correlation of nutrient release rate with mass loss rate of bald cypress (A), Chinese willow (B), and heterogeneous-leaf (C) treatments (n=40).

| Foliar type | C% | N% | P% |
| --- | --- | --- | --- |
| A | 0.946** | 0.969** | 0.928** |
| B | 0.946** | 0.953** | 0.940** |
| C | 0.972** | 0.963** | 0.963** |

* and ** indicate significance at the 0.05 and 0.01 levels, respectively.

**Figure S1.** Mass loss of bald cypress (A), Chinese willow (B), and heterogeneous-leaf (C) treatments under different submergence depths during the decomposition process.

**, P *<* 0.01 and *, P < 0.05. The species, time, and their interactions were all **. CK: control check (no submergence); SS: shallow submergence; DS: deep submergence.

**Figure S2.** Nutrient (C, N, and P) release of bald cypress (A1, A2, A3), Chinese willow (B1, B2, B3), and heterogeneous-leaf (C1, C2, C3) treatments under different submergence depths during the decomposition process.

CK: control check (no submergence); SS: shallow submergence; DS: deep submergence.

**Figure S3.** Changes in air temperature, water temperature, and dissolved oxygen of the reservoir during the experimental period.

SS: shallow submergence; DS: deep submergence.
